# An update on the incidence of human giardiasis in Scotland, 2011–2018

**DOI:** 10.1186/s13071-020-04160-9

**Published:** 2020-06-08

**Authors:** Lynne C. Ferguson, Alison Smith-Palmer, Claire L. Alexander

**Affiliations:** 1 Scottish Microbiology Reference Laboratories, Glasgow, Scotland, UK; 2 Health Protection Scotland, Glasgow, Scotland, UK

**Keywords:** *Giardia*, Scotland, Under-reporting, Incidence

## Abstract

**Background:**

*Giardia duodenalis* is one of the most common parasites in the UK to cause diarrhoeal illness. Giardiasis is likely to be significantly under-reported in the UK as laboratory testing is largely based on examining stool samples from individuals with a recent travel history. This results in the majority of locally-acquired cases going undetected. To increase awareness of giardiasis, we describe data gathered from cases reported within Scotland during 2011–2018.

**Methods:**

All of the 21 Scottish National Health Service (NHS) diagnostic microbiology laboratories performed microscopy examination to detect *Giardia* cysts in stools, from mostly travel-related cases. The exception was one laboratory that implemented an antigen-based enzyme immunoassay in 2015. This resulted in every submitted stool being tested for *Giardia*. Laboratory-confirmed cases of giardiasis were reported to Health Protection Scotland (HPS) *via* the Electronic Communication of Surveillance in Scotland (ECOSS) during the eight-year period. Data for calculating the incidence per 100,000 of the population were obtained from the National Records of Scotland mid-2018 population estimates in Scotland.

**Results:**

A total of 1631 Scottish cases were reported during 2011–2018 (8-year mean: 204; range: 166–269). National Health Service Grampian, Borders and Lothian reported the highest incidence of *Giardia* (9.8, 7.5 and 6.7 per 100,000, respectively), all of which were above the Scottish mean incidence (3.8 per 100,000). Following the implementation of antigen testing in NHS Grampian during 2015, reports significantly increased 3.6-fold (*P* = 0.005). The highest incidence of giardiasis occurred in the 20–49 years age group (mean 5.4 per 100,000). Of interest, the mean incidence of giardiasis was significantly higher in males than in females (4.8 *versus* 3.1 per 100,000, respectively; *P *< 0.0001).

**Conclusions:**

This report highlights the need to capture enhanced information on every laboratory-confirmed case of giardiasis to gain a better understanding of the local sources and transmission pathways occurring in Scotland. In addition, implementing sensitive, automated technologies across UK NHS diagnostic microbiology laboratories to permit the efficient, routine testing of every submitted stool for *Giardia*, should be encouraged to ensure all cases are identified and treated appropriately.
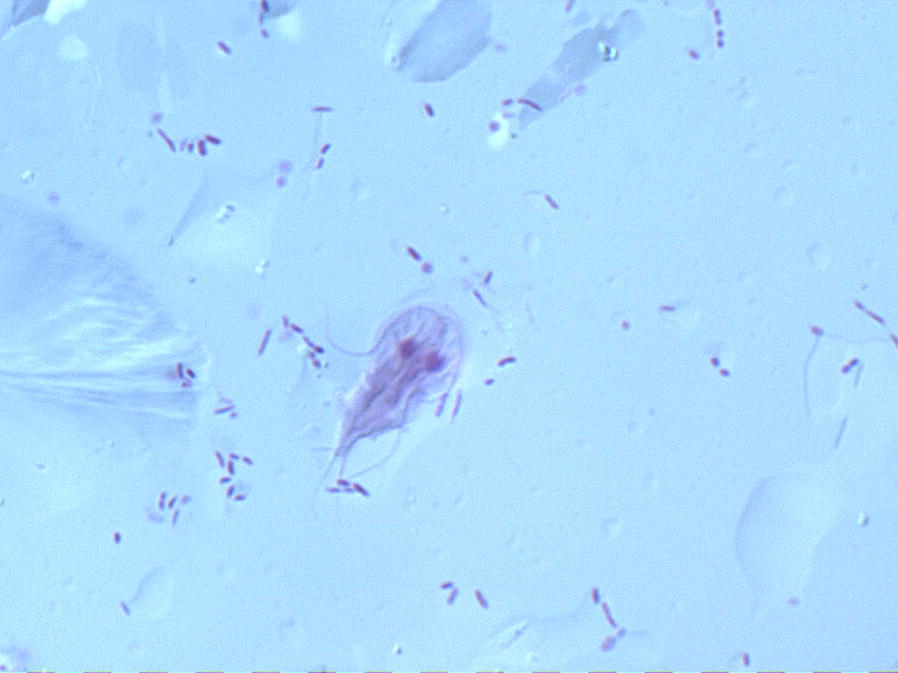

## Background

*Giardia duodenalis* (syns *Giardia intestinalis* and *Giardia lamblia*) is a flagellated protozoan found worldwide and is one of the most common parasitic causes of gastrointestinal illness in the UK [1]. Giardiasis commonly presents as foul-smelling diarrhoea, abdominal pain, and excessive flatulence. Infection can result in long-term issues including irritable bowel syndrome (IBS) and chronic fatigue [2]. Asymptomatic carriage is also possible [3]. Of importance, giardiasis is a treatable disease requiring the administration of antimicrobial drugs such as metronidazole and tinidazole [4]. Transmission occurs *via* the faecal-oral route through the ingestion of infective cysts. While human to human transmission occurs, zoonotic transmission remains debatable [5, 6]. Outbreaks of *Giardia* associated with contaminated food and water sources have been reported [7, 8].

Stools are submitted for *Giardia* testing to National Health Service (NHS) diagnostic microbiology laboratories from general practitioners (GPs) and hospital clinicians throughout Scotland. Requests are mostly received from individuals with a recent travel history. Samples may also be referred when an individual is stated to have had a previous episode of giardiasis or is immunocompromised. Testing is guided by the UK Standards for Microbiology Investigations (SMI) documentation of which there are three relating to parasite investigations (SMI B30, B31 and S7) [9]. Laboratories use this guidance to develop local testing algorithms to select appropriate samples for *Giardia* investigation. Despite increasing evidence that *Giardia* is acquired within the UK [10], an audit of Scottish NHS diagnostic microbiology laboratories found that, based on the UK SMI guidance, the majority of stools tested for *Giardia* were those from individuals who had recently travelled outside the UK [11]. This results in less than 20% of submitted stools being examined for *Giardia*. Use of commercial molecular or antigen-based assays have been reported to the permit screening of large numbers of stools for *Giardia* (and other gastrointestinal pathogens) [12–14]. Despite alternative methods being available, most Scottish NHS diagnostic microbiology laboratories use microscopy to detect *Giardia* [11]. These factors, combined with asymptomatic carriage, are likely to result in the significant under-ascertainment and under-reporting of cases [15].

This report raises awareness of *Giardia* by describing surveillance data captured for laboratory-confirmed cases of *Giardia* in Scotland over an 8-year period (2011–2018). At present, there is a drive for laboratories in the UK to move towards testing a wider selection of stools for *Giardia* and to implement sensitive, automated detection methods. These changes will greatly impact on the numbers of reported cases in Scotland in the future.

## Methods

As *Giardia* is notifiable under the Public Health (Scotland) Act 2008, details of laboratory-confirmed cases were shared with NHS Health Protection Teams for follow-up and action [16]. This information was also shared with the Electronic Communication of Surveillance in Scotland (ECOSS) to Health Protection Scotland (HPS) for national surveillance. Reports of every laboratory-confirmed case of giardiasis during 2011–2018 were collected from 21 NHS diagnostic microbiology laboratories within 14 territorial Scottish NHS health boards. At the time of data collection, all except one laboratory performed microscopy of wet mount preparations without the addition of stains to detect *Giardia* cysts and trophozoites. The exception was a laboratory in NHS Grampian that changed from performing microscopy to implementing an enzyme immunoassay (EIA) antigen detection method (*Giardia*/*Cryptosporidium* Combo EIA kit, IVD Research, Carlsbad, CA, USA) in 2015 [17].

Microsoft Excel and SPSS (version 21.0, SPSS, Inc., Chicago, IL, USA) were used for data analysis. The incidence of giardiasis was calculated per 100,000 by; NHS health board, age-band and sex over the eight-year period. The mean incidence per 100,000 in Scotland was also determined. In addition, incidence per 100,000 was calculated for NHS Grampian data in isolation for the 8-year period, as it was the only health board to test every stool for *Giardia* at the time of data analysis. Population data for calculating incidence was obtained from the National Records of Scotland mid-2018 population estimates in Scotland [18]. Seasonality was reported as the mean number of laboratory-confirmed cases rather than the incidence, as the population numbers for individual months were not available.

The Z-test was used to compare the differences in the mean incidence of giardiasis by sex and following a change in laboratory testing method in NHS Grampian. A probability (*P*)-value< 0.05 was considered statistically significant. Whilst it is rare to receive more than one sample from an individual, an episode criterion of four-weeks was applied to ensure multiple samples from the same individual were only counted once.

## Results

### Incidence by NHS Health Board

**Fig. 1:**
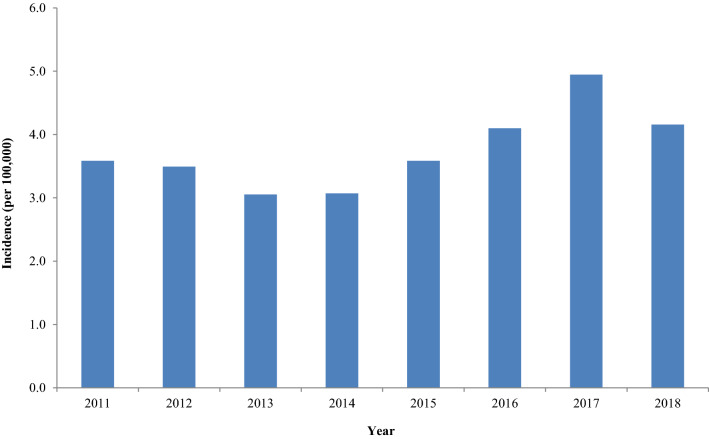
Incidence of laboratory-confirmed giardiasis cases per 100,000 of the population in Scotland, 2011–2018

Between 2011 and 2018, a total of 1631 cases were reported in Scotland (mean: 204; range: 166–269) with the lowest incidence reported during 2013 and 2014 (3.1 per 100,000) (Fig. 1). The highest incidence occurred during 2017 (4.9 per 100,000) (Fig. 1). The mean incidence over the 8-year period was 3.8 per 100,000.

**Fig. 2:**
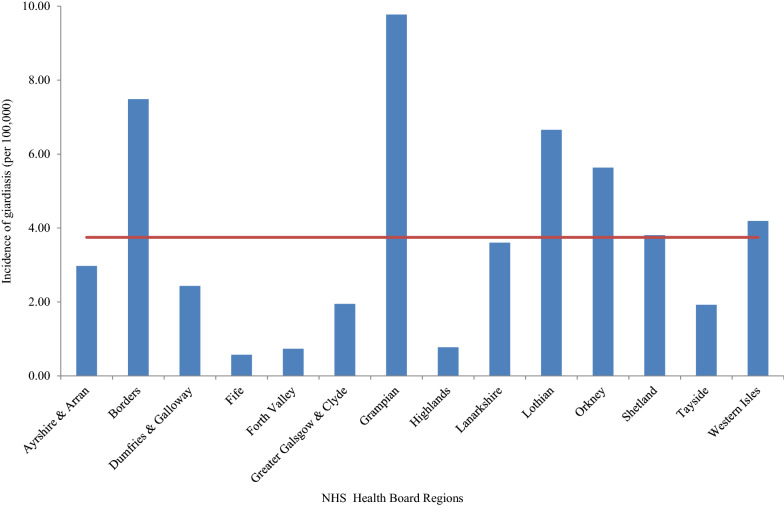
Incidence of laboratory-confirmed giardiasis cases per 100,000 of the population by NHS health board, 2011–2018. The solid line represents the mean incidence in Scotland

By individual NHS health board, Grampian, Borders and Lothian reported the highest incidence (9.8, 7.5 and 6.7 per 100,000, respectively; Fig. 2). Due to the small population size in Orkney, Shetland and the Western Isles, their high incidence of giardiasis should be viewed with caution.

### NHS Grampian

**Fig. 3:**
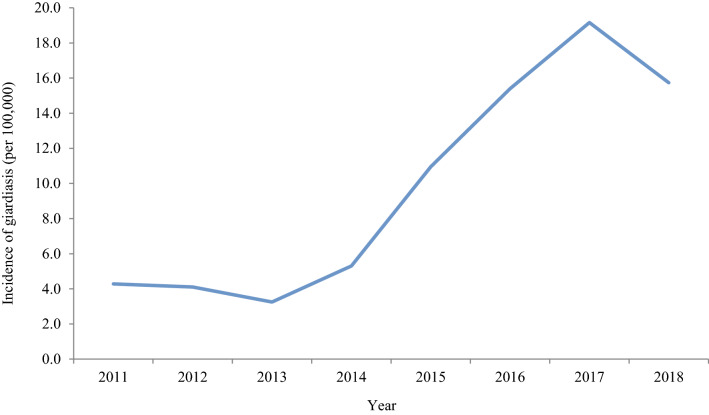
Incidence of laboratory-confirmed giardiasis cases per 100,000 of the population within NHS Grampian, 2011–2018

*Giardia* reports from the NHS Grampian health board were examined further to explore the impact of changing the laboratory testing method during 2015. The change from microscopy to the use of an EIA to screen every stool sample for *Giardia* resulted in a significant 3.6-fold increase in the mean incidence of giardiasis from 4.2 (2011–2014) to 15.3 (2015–2018) per 100,000 (*P* = 0.005; Fig. 3).

### Age and sex distribution

Overall, the incidence of giardiasis was significantly higher among males (mean 4.8 per 100,000) than females (mean 3.1 per 100,000) (*P *< 0.0001).

**Fig. 4:**
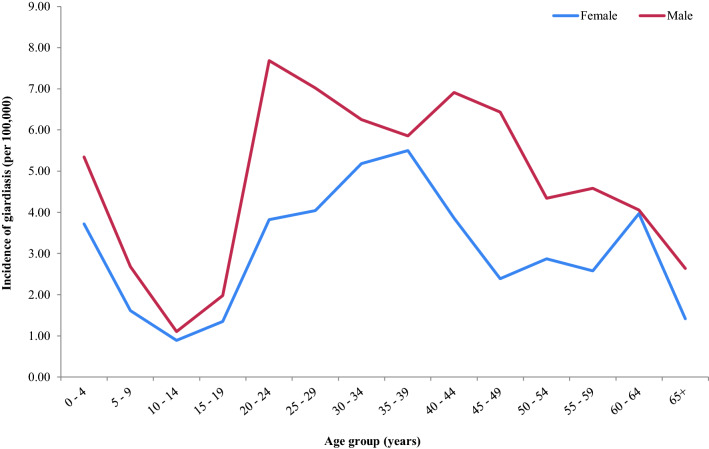
Incidence of laboratory-confirmed giardiasis cases per 100,000 of the population in Scotland during 2011–2018 by age and sex group (NB: two reports were missing data on sex)

In males, incidence of giardiasis started high in the 0–4 age group (5.4 per 100,000) then decreased between the ages of 5–19 (Fig. 4). In the 20–24 years age group, the incidence increased sharply (7.7 per 100,000) and remained high then gradually declined from age 50 years onwards reaching an incidence of 2.6 per 100,000 by age 65 years and beyond (Fig. 4).

Incidence of giardiasis in females aged 0–4 also began high (3.7 per 100,000) then decreased between the ages of 5–19 (Fig. 4). Incidence rose gradually from age 20, peaking in the 35–39 age group (5.5 per 100,000) (Fig. 4). Thereafter, incidence declined in females except in those aged 60–64 years in which the incidence of giardiasis increased (4.0 per 100,000) (Fig. 4).

### Seasonality

The number of laboratory-confirmed cases per month remained relatively stable throughout the 8-year period (mean 17 per month). The lowest mean number of cases was reported during February (*n* = 12) and the highest was reported during August (*n* = 26).

## Discussion

The present data raise awareness of giardiasis by exploring the incidence in Scotland over an 8-year period. To our knowledge, this is the first Scottish report to describe a significantly higher incidence of giardiasis in males than females which merits further exploration. It also highlights preliminary findings to demonstrate the impact on the number of reported cases when sensitive, automated laboratory methods are applied.

The 3.6-fold increase in incidence within NHS Grampian during 2015–2018 was largely influenced by a change in testing method within one laboratory. In 2015, antigen testing was introduced to replace microscopy. This permitted the screening of every submitted stool sample for *Giardia*. The data suggest that, in addition to *Giardia* being associated with travel outside the UK, it is also acquired locally. Further supportive evidence in the UK is described in a case-controlled study carried out in North-West England, where the authors highlight that as many as 75% of giardiasis cases were locally acquired [10]. As most Scottish laboratories currently use microscopy to detect *Giardia* and only test for it in 2–18% of submitted stools, it is highly likely that this pathogen is under-reported and many of the locally-acquired cases will go undiagnosed [11, 17]. The implementation of molecular testing for stool pathogens using assays including the BioFire FilmArray gastrointestinal panel (Biomerieux, Marcy l’Etoile, France), EntericBio Gastro Panel 2 (GP2; Serosep, Limerick, Ireland) and Luminex xTAG gastrointestinal pathogen panel (GPP; Luminex Corporation, Toronto, ON, Canada) is gradually being introduced across the UK and elsewhere [12, 13]. Molecular panels for the diagnosis of gastrointestinal parasites permit the rapid screening of a large number of stools for *Giardia* and reduce the need for microscopy expertise. However, molecular tests may not be cost-effective in every laboratory setting. In addition, deoxyribonucleic acid (DNA) may still be present soon after an infection has cleared and therefore, detection of DNA does not always represent an active current infection. Inhibitors may also be present in stools that impact on downstream molecular testing, which may lead to false negative results.

The exact reservoirs of *Giardia* in Scotland remain unclear. Of interest, rural and semi-rural areas in Scotland include the Borders and Grampian regions, which have the highest incidence of giardiasis. This coincides with having higher numbers of private water supplies than other regions and these are not subjected to strict monitoring procedures. Therefore, it is reasonable to suggest that individuals are at greater risk within these regions due to exposures from potentially contaminated water sources. In addition, grazing livestock within these rural/semi-rural regions may provide greater opportunities for acquiring infection through increased exposure to infective animal stools. Livestock and companion animals have been suggested as reservoirs as they share the same molecular types, known as assemblages, as those identified in humans (assemblages A and B). One UK study identified *Giardia* from 44% (28/64) of sheep examined and from 33% (93/283) of cattle [5]. However, assemblage E, which has not been identified in the human host, was found to be predominant. Despite these findings, assemblage A was also identified from 17% of sheep (4/24) and from 25% of cattle (16/63) in this study, suggesting the potential for zoonotic transmission. Data on companion animals in the UK are limited to one study on 878 dogs within kennels where mostly assemblages C and D were identified from 40 of the 41 samples that were genotyped, with only one sample found to be assemblage A [6]. In contrast, a Spanish study in dogs (*n* = 348), which included pet dogs within homes as well as dogs in kennels, found that 57% (20/35) of samples were either assemblage A, B or mixed (including A and/or B) infections [19]. To explore zoonotic potential further, robust in-depth molecular profiling is required to identify *Giardia* assemblages and how these relate to reported exposures from humans, companion animals, livestock, wildlife and the environment.

Unlike *Cryptosporidium*, a parasite which has well-described seasonal peaks occurring in spring and autumn [20, 21], *Giardia* numbers remain relatively constant throughout the year with an increase in August. However, due to the bias towards testing mostly travel-related samples, the difference is likely to reflect increased travel abroad and greater outdoor exposures during the warmer weather.

Incidence of giardiasis was highest in two age groups; 0–4 and 20–49 years-old, with a higher incidence reported in the latter group. Previously published Scottish surveillance data from 1988–2003 also showed a high incidence in young children (0–4 years-old) and adults (20–39 years-old) [22]. However, in contrast, this earlier report described more cases within the 0–4 age group in comparison to the 20–39 age group, highlighting a shift across the years. The higher incidence in young children may reflect the healthcare seeking behaviour of parents and the increased sample submissions by clinicians. There are also likely to be a variety of other factors to explain this, including poorer hygiene behaviour in young children, the lack of previous exposure to *Giardia*, an immature immune system, increased exposure to recreational water, adoption from overseas and close contact with other children and family members. In one study that examined household contacts of infected individuals, having children under five years in the household was found to be a significant risk factor [15]. The authors also highlighted the presence of asymptomatic infection in 37 out of 41 infections identified from 212 household members. Therefore, transmission between adults and young children is likely to be underestimated.

Of interest, incidence of giardiasis in males was significantly higher than in females within every age group, particularly amongst adults. Sexual practices may account for spread within certain age groups and this has been reported in the MSM community [23]. However, this does not explain the higher incidence in other age groups. It has been suggested that females possess better hand hygiene practices and knowledge [24]. This, along with environmental exposures and/or occupational hazards, could contribute towards the higher incidence of giardiasis reported in males. This finding warrants further investigation as it may involve more complex gender factors.

## Conclusions

How many giardiasis cases within Scotland and the rest of the UK go undetected remains unknown. Despite being identified within every Scottish NHS health board, the true number of symptomatic cases will only be revealed when all diagnostic laboratories progress to test every submitted stool for this pathogen. To support this, the implementation of sensitive, automated technologies within NHS diagnostic microbiology laboratories should be encouraged. By identifying travel-related and locally-acquired cases, clusters and outbreaks will be revealed that have previously gone undetected. Robust information to explain the gender bias and to define local reservoirs and transmission routes within Scotland is currently lacking. To address these points, the Scottish Health Protection Network (SHPN) has established an enhanced surveillance programme for *Giardia* to capture vital, in-depth information from individual cases within specific Scottish NHS health boards. This, combined with improvements to laboratory detection methods, better molecular tools for outbreak investigations and wider stool selection protocols, are absolutely essential to gain a better understanding of giardiasis. It is crucial individuals are tested and treated to prevent local and widespread outbreaks of this neglected pathogen.

## Data Availability

Data supporting the findings of this article are included within the article.

## Abbreviations

NHS: National Health Services
HPS: Health Protection Scotland
ECOSS: electronic communication of surveillance in Scotland
IBS: irritable bowel syndrome
GPs: general practitioners
SMI: standards for microbiology investigations
EIA: enzyme immunoassay
MSM: men who have sex with men
SHPN: Scottish Health Protection Network
